# A SWOT Analysis of Pharmacy Students’ Perspectives on e-Learning Based on a Narrative Review

**DOI:** 10.3390/pharmacy11030089

**Published:** 2023-05-22

**Authors:** Carla Pires

**Affiliations:** CBIOS—Research Center for Biosciences & Health Technologies, University Lusófona, Campo Grande 376, 1749-024 Lisbon, Portugal; p5558@ulusofona.pt

**Keywords:** SWOT analysis, e-learning, education, students’ perspectives, pharmacy, students’ well-being

## Abstract

Background: Online education became the new normal during the COVID-19 pandemic. However, the number of studies exploring the potential advantages/disadvantages of e-learning in pharmacy courses is limited. Study aim: to propose a strengths, weaknesses, opportunities, and threats (SWOT) analysis of e-learning according to pharmacy students’ perspectives. Methods: A narrative review was conducted to examine student pharmacist perspectives on e-learning. Results: Diverse strengths and weaknesses (internal environment) and opportunities and threats (external environment) were identified, which were grouped into categories, such as (1) students’ well-being (e.g., access to classes anywhere vs. students’ psychological or physical disorders); (2) teachers and materials (e.g., more diverse/interesting audiovisual materials vs. too challenging materials); (3) technologies (e.g., new education strategies, such as gamification vs. barriers in the access to the internet); (4) classes/training (e.g., more versatile/immediate classes vs. eventual presence of other persons during online classes); and (5) faculty/school of pharmacy (e.g., availability of technical support). Conclusion: Overall, online education seems to be suitable for pharmacy students, although diverse challenges should be addressed, such as the well-being of students or lack of standards. Pharmacy schools should regularly identify/define and implement measures to reinforce opportunities and strengths as well as to solve threats and weaknesses.

## 1. Introduction

Pharmacists are health professionals responsible for preparing, dispensing, and re-viewing therapy, providing pharmaceutical consultations, advising patients, and developing new medicines or implementing and validating clinical or genetic analyses, among other functions [1,2]. Both the World Health Organization (WHO) and International Pharmaceutical Federation (FIP) advocate that pharmacy students need to be prepared to address future digital health patients’ needs, such as the provision of telehealth consultations with patients. Consequently, healthcare students need to develop new skills to use online health tools or to orientate patients on the use and comprehension of digital technologies, with e-learning methodologies facilitating the teaching and acquisition of these skills [3,4].

E-learning commenced long ago, with one of the first reports about online education at the University of Illinois, USA, in 1960 [5]. Before the COVID-19 pandemic, online pharmacy education was already successfully and routinely applied to provide continuing education, with high rates of pharmacy students’ acceptance [6]. The COVID-19 pandemic was declared by the World Health Organization on 11 March 2020, with a global outbreak of coronavirus, an infectious disease caused by the severe acute respiratory syndrome coronavirus 2 (SARS-CoV-2) [7]. Because of the COVID-19 outbreak, there was a shift from face-to-face teaching to online teaching, due to the successive lockdowns imposed by the health authorities at a global level. Healthcare teachers, such as pharmacists, nurses, or physicians, were also forced to cancel face-to-face classes and adopt online teaching [8].

Several benefits and/or problems related to teaching pharmaceutical sciences have been identified during the COVID-19 pandemic. The benefits of online teaching were as follows: being more comfortable, spending less time in transport, and having more time for family, among others [9,10]. However, several problems related to online teaching were also identified, such as spending too much time on screens, less communication/interaction with colleagues and teachers, more anxiety and mental problems, less knowledge acquisition, more difficulty in consulting/understanding study materials, less acquisition of new practical/laboratorial skills, and more distractions at home [11,12].

Laboratorial and practical classes are common in pharmacy studies. However, the provision of online lab classes is complex to implement and operate [1,2]. Practical and laboratory classes were canceled or adapted for online teaching in health courses during the COVID-19 pandemic, with a likely reduction in the acquisition of new laboratory skills by students [9].

Only about half of the pharmacy students (around 5000 students) perceived online teaching as more useful/favorable than in-person classes in a recent systematic review aiming at characterizing the perception of pharmacy students on online teaching during the COVID-19 pandemic. This systematic review concluded that online teaching needs to be regularly monitored and optimized. Positively, pharmacy students expressed an interest in maintaining online learning in future [13]. For instance, students expressed interest in the use of social networks (e.g., Facebook) in e-learning, as they are based on human interactions between colleagues and/or teachers [13,14].

The elements of SWOT analysis are strengths, weaknesses, opportunities, and threats. By definition, a SWOT analysis “helps find the best match between environmental trends (opportunities and threats) and internal capabilities (strengths and weaknesses)” [15,16]. SWOT analyses are used as planning tools, which can be applied in a strategic planning project or, merely, to understand an organization or a certain situation. Particularly, SWOT analysis can be used to generate ideas and solutions, to solve problems, to plan, to support personal development, to make decisions, or in the scope of workshop sessions as a planning tool, among others [17,18].

Here, a SWOT analysis is proposed to investigate and understand online teaching in pharmacy education. Additionally, a narrative review was adopted to carry out the present work. Narrative reviews comprehensively cover a specific topic. These reviews include the compilation and evaluation of relevant information through the convenient selection of pertinent works/papers [19,20]. Only one paper related to the present topic has been identified [18], which seems to confirm an existing gap in the state of the art regarding the elaboration of SWOT analysis of pharmacy students’ perspectives on online education. According to the findings from this previous SWOT analysis of a specific in-service and e-learning course for pharmacists working in a public health system, the success of continuous education projects through e-learning was influenced by the course’s internal elements (e.g., contents, organization, and didactic methods) and external elements from the healthcare institutions (e.g., the work conditions of health professionals, accessibility to technological resources, and the political and economic scenario) [18].

Thus, the study objective was to propose a SWOT analysis of e-learning according to pharmacy students’ perspectives based on a narrative review. Additionally, the following research question was defined: How can the created SWOT analysis contribute to the optimization of virtual pharmacy courses?

## 2. Materials and Methods

A narrative review was carried out following the recommendations of Gasparyan et al. (2011) [19].

### 2.1. Searched Databases

The searched databases were as follows: PubMed, SciELO, Google Scholar, and sites of known international organizations, such as the World Health Organization (WHO) and International Pharmaceutical Federation (FIP).

### 2.2. Keywords and Covered Timeframe

All keywords were conveniently selected based on their connection with the present topic. The searched strings of keywords were as follows: (e-learning or online education) and pharmacy and (students or undergraduates). Only the published papers (or other relevant documents) in the last three years were selected (1 January 2019 to 15 January 2023).

### 2.3. Search Methodology and Data Extraction

The strings of keywords were introduced in the databases or websites. Data were collected during January 2023 (last search on 15 January 2023). The same researcher conducted the search, selected the papers and other relevant documents/guidelines, and summarized the key findings. Papers (and other documents) were conveniently selected based on their pertinence and contribution to the present topic. Strengths, weaknesses, opportunities, and/or threats were identified and extracted by one author. Results were collected/organized in a tabular format (a Word document).

All study findings were double-checked for accuracy at the end. For instance, data were checked in the steps of (i) data collection and (ii) elaboration/drafting of discussion. The extraction of data followed a saturation methodology, i.e., the search was previously defined as concluded when the sequential analysis of 5 new works/papers did not result in any new output. All searches and data collection were manually carried out.

#### Previous Research on the Present Topic

A previous review (narrative, systematic, or meta-analysis) concerning the present topic was not identified in PubMed, Google Scholar, or SciELO on 1 January 2023 (i.e., the proposal of a SWOT analysis of e-learning according to pharmacy students’ perspectives based on a narrative review). The searched keywords were SWOT and pharmacy and (online education or e-learning). A second search was carried out with the keywords (SWOT and pharmacy and student). In particular, the search options review and systematic review were activated in PubMed. A related research paper was identified in Google Scholar [18], but it was not a review. These facts seem to confirm the relevance of the present study.

### 2.4. Inclusion and Exclusion Criteria

Inclusion criteria were as follows: peer-reviewed papers or other documents (e.g., guidelines) describing pharmacy students’ perspectives on e-learning. E-learning is here defined as any methodology adopted to provide online education. Pharmacy students’ perspectives are defined as any students’ opinions/viewpoints about e-learning. All types of papers were classified as eligible to be included, such as reviews, original research, and commentaries. Exclusion criteria were as follows: other topics or papers out of the covered timeframe.

### 2.5. SWOT Analysis

A SWOT analysis of pharmacy students’ perspectives on e-learning was constructed. The following definitions were considered [15,16]:

- Strength—a resource or capacity related to pharmacy students’ perspectives on e-learning, regarding the organization/school (internal environment).
- Weakness—a limitation, a fault, or a defect related to pharmacy students’ perspectives on e-learning, regarding the organization/school (internal environment).
- Opportunity—a favorable situation related to pharmacy students’ perspectives on e-learning, regarding the organization/school, i.e., a trend/change or overlooked need that could lead to improvement if the need is met (external environment).
- Threat—a barrier, a constraint, or anything external that might cause problems, damage, or injury related to pharmacy students’ perspectives on e-learning, regarding the organization/school (external environment).

#### Thematic Analysis

Considering the expected heterogeneity of the emerging topics from the SWOT analysis (see Table 1), all identified topics were classified into groups/subcategories. These groups/subcategories were conveniently created based on a content analysis of the identified topics (i.e., strengths, weaknesses, opportunities, and threats).

### 2.6. Quality Assessment of the Present Narrative Review and of the Selected Papers/Works

The quality evaluation of the present narrative review was carried out according to SANRA—a scale for the quality assessment of narrative review articles [20]. Only peer-reviewed papers were selected, which constituted the only quality indicator. The works/documents from known international organizations were previously assumed to be acceptable since these documents are usually developed by recognized international experts.

## 3. Results

### 3.1. Selected Papers/Documents

The total numbers of papers/documents identified on 15-1-2023 were as follows: PubMed (n = 65 for “e-learning and pharmacy and students”; n = 11 for “e-learning and pharmacy and undergraduates”; n = 40 for “online education” and pharmacy and students; n = 9 for “online education” and pharmacy and undergraduates); SciELO (n = 1 for “e-learning and pharmacy and students”; n = 0 for “e-learning and pharmacy and undergraduates”; n = 0 for “online education” and pharmacy and students; n = 0 for “online education” and pharmacy and undergraduates); Google Scholar (around 47,000 outputs/works for all strings of keywords); and the sites of known international organizations, such as the World Health Organization (WHO) (n = 0 for all strings of searched keywords) and International Pharmaceutical Federation (FIP) (n = 1).

The displayed papers/works from each searched database were not all read/analyzed; the extraction of data followed a saturation methodology, i.e., the analysis of the selected papers/works was previously defined as concluded when the sequential analysis of five new works/papers did not result in any new output for the SWOT analysis.

Globally, 39 papers and 1 report were selected: PubMed (n = 33) [10,13,21,22,23,24,25,26,27,28,29,30,31,32,33,34,35,36,37,38,39,40,41,42,43,44,45,46,47,48,49,50,51], SciELO (n = 1) [52], Google Scholar (n = 6) [8,53,54,55,56,57], and sites of known international organizations (n = 1) [3].

The selected publications were classified as follows: 7 reviews [8,13,23,41,43,52,53]; 31 original research publications [10,21,22,24,25,26,27,28,29,30,31,32,33,34,35,36,37,38,39,40,42,44,45,46,48,49,50,51,55,56,57]; 1 commentary [47]; 1 case study [54]; 1 report [3].

### 3.2. SWOT Analysis

The proposed SWOT analysis of e-learning from the students’ perspective is presented in Table 1. Information was extracted from the selected papers/documents.

**Table 1:** SWOT analysis of e-learning from the students’ perspective.

STRENGTHS—Internal Environment [3,8,10,13,21,22,23,24,25,26,28,29,30,32,33,35,36,37,38,39,40,41,42,43,44,45,46,47,48,50,51,52,53,55,56,57]	WEAKNESSES—Internal Environment [8,13,21,22,23,26,27,39,43,44,47,48,53,54,55,57]
Competency and confidence in online health practices. Improved concentration. Students’ engagement, acceptance, motivation, satisfaction, or positive perceptions. Improved acquisition of knowledge and skills. Potential to build their knowledge or acquire new digital skills in pharmaceutical online consultations. Improved students’ autonomy and independent learning. Reinforced skills, problem solving, critical thinking, and adaptability. Satisfaction with online education, including the examination platform, and online examinations. Intellectually challenging. Enhanced knowledge, competencies, and skills, regarding digital technologies. Satisfying placement experiences (e.g., virtual consultations). Offer of clinical practice scenarios in a safe and controlled environment. Application of previously acquired computer and technological skills. Quality of materials and teachers’ performance (e.g., covered topics). Interactive materials and e-lectures seem to be attractive. Videos are helpful in the preparation of laboratory/practical classes. Access to additional health information. Use of new technologies, such as artificial intelligence (AI) in industrial pharmacy settings or digital health in pharmaceutical care. Digital interactivity, such as through mobile applications, augmented reality, gamification, or social networking. Innovative study methodologies/practices. Flexible schedules, possibility of saved time and time management. Versatility, such as in giving feedback, continuing a previous classroom lesson, or being enrolled in specific training sessions. Class/session readiness. Sessions recorded online can be useful for study enforcement. Possibility of more flexible programs. Offer of diverse learning resources, such as clinical platforms or a laboratory to test digital health. Satisfaction with the diversity and applicability of diverse learning resources. Extended online feedback, such as automatic scoring by teachers/students. Internal available technical support. Opportunity of receiving online interprofessional education. Versatile and applicable in the case of postgraduation training, continuing education, or internships. Development, evaluation, and validation of online education by internal organizations and stakeholders, including students.	Fewer interactions or inability to network with peers, experts, and teachers. Less in-person patient care experience, with some students reporting difficulties in acquiring clinical skills. Lack of motivation or less positive attitudes. Limitations in the development of social skills. Need to develop new communication skills. Lack of past experience in using online tools. Limited digital literacy and technology experience in some countries. Cheating in online assessments. Limited conditions for providing laboratory and practical classes. Restricted acquisition of laboratorial and practical skills and competencies. Some digital training experiences may not improve students’ knowledge, motivation, interest, or positive perception (negative experiences). Too challenging e-learning materials. Lack of instructions or subjective norms. Online examination platforms may not be appropriate for all students. Lack or limited evaluation of student performance outcomes. Hardware, software, and internet are needed. Internet issues: speed, breakdown, or stability. Avoiding the use of common social networking tools such as YouTube, Twitter, Instagram, or Facebook. Lack of electronic devices such as smartphones or tablets. Too challenging and complex e-learning tools; low usability of the e-learning system (ease of use). Lack of adequate hardware and software assistance in schools/faculties.
**OPPORTUNITIES—External Environment** [33,44,48,55]	**THREATS—External Environment** [8,13,21,22,27,31,34,44,47,49,54,55]
Location flexibility. Spending less time traveling, and reduction in costs. More economic and sustainable. Improved family conviviality, comfort, and satisfaction. Accessibility of information everywhere, 24 h/day. Chance to receive effective and timely support from a teacher or other academic staff. Chance to receive external training.	Higher risk of psychological problems, anxiety, stress, depression, low resilience, loneliness, social isolation, and frustration. Missing interactions with colleagues outside of classes. More potential for health problems (e.g., physical posture and vision issues). Increased smoking, and an augmented consumption of less healthy foods or caffeinated beverages. More likely to be distracted at home. Absence of a suitable private space for classes, teleconsultation, or other clinical interactions. Problems related to the use of software, hardware, net failure, or other technical issues. High internet and technological costs. Eventual compromise of confidentiality of data (e.g., cybersecurity issues). Not having internet access or a laptop at home.

#### Thematic Analysis

The defined groups/subcategories were conveniently defined as follows:

- Strengths: (i) students, (ii) materials and teachers, (iii) technologies, (iv) online classes/training, and (v) faculty/school of pharmacy and other variables;
- Weaknesses: (i) students—human interactions and communication, (ii) students—other, (iii) classes, and (iv) technologies;
- Opportunities: (i) students and (ii) online classes;
- Threats: (i) students’ well-being, (ii) classes, and (iii) technologies.

### 3.3. Quality Assessment of the Present Narrative Review and of the Selected Papers/Works

The present narrative review was classified as compliant with the requisites of the SANRA scale, with the achievement of the maximum score (12 points) in all evaluated dimensions: (1) justification of the article’s importance for the readership; (2) statement of concrete aims or formulation of questions; (3) description of the literature search; (4) referencing; (5) scientific reasoning; and (6) appropriate presentation of data [20]. Moreover, none of the selected papers was excluded due to quality issues, with all selected papers being peer-reviewed.

## 4. Discussion

To ensure a structured argumentation, the discussion is organized as follows: strengths (4.1), weaknesses (4.2), opportunities (4.3), threats (4.4), reply to research question and practical implications (4.5), and limitations and strengths (4.6). This paper is one of the first SWOT analyses and narrative reviews focused on e-learning from student pharmacists.

### 4.1. Strengths

Overall, students pointed out diverse strengths regarding the internal environment of pharmacy schools. These strengths were organized into five topics: (1) students; (2) materials and teachers; (3) technologies; (4) classes/training; and (5) faculty/school of pharmacy and other variables.

#### 4.1.1. Students

In general, pharmacy students declared improved feelings, such as self-confidence, acceptance, engagement, satisfaction, motivation (e.g., hedonic motivation), intellectual development, and security in relation to patient interactions (e.g., virtual patients) or a positive general perception about online education [8,13,21,39,43,57]. Students declared being satisfied with the possibility of applying previous computer or other technological skills (e.g., “I have satisfactory computer skills for dealing with online course/assignments”) [48], and students recognized the usefulness of e-learning (e.g., “My attention to the class tasks during e-learning session was greater in comparison to the traditional face-to-face class meetings”) [24]. Additionally, some pharmacy students achieved or declared better skills or competencies, such as enhanced concentration, knowledge acquisition, autonomy (students’ centered process), digital skills, problem solving, critical thinking, and quicker adaptation to new working scenarios [25,44,52,53,55].

#### 4.1.2. Materials and Teachers

Visual aids, videos, and interactive materials were shown to be attractive materials/documents for pharmacy students. For instance, audiovisual enhanced illustrations encouraged students to interact with the lecture material prior to the virtual class [24,44,46]. Additionally, it was possible to have access to information in different formats, such as patient digital health records or digital biomarkers, which may lead to the study of new relevant topics in pharmacy practice [56]. Students’ satisfaction with the quality of developed materials (e.g., type of covered topics—more or less interesting or too challenging e-learning materials) and/or teachers’ performance was evaluated and reported in a limited number of studies [38,46,48].

Considering that digital health education is widely used at a global level, disruptive teachers capable of using innovative technologies and e-learning methodologies are needed in the future of pharmacy education [47]. For instance, flipped e-learning activities could draw pharmacy students’ attention, motivate students, increase fun, encourage students to prepare lectures, improve concentration, raise the level of interactions, and provide more time for activities and exercises during the class [46].

#### 4.1.3. Technologies

Besides the internet, IT infrastructures, laptops, tablets, and mobile phones, other devices may be required (e.g., for augmented reality) [3]. Many students prefer smartphones for online access, but tablets and laptops were also satisfactorily used [22,30].

The gradual translational rotation to a new technological environment at pharmacy schools, necessarily, implies the development of innovative study methodologies and students’ adaptation. Examples regarding clinical pharmacy and telemedicine include active learning sessions or digitally adapted games, virtual patient software, augmented reality, chronic disease management, and medication review through software applications [32,33].

Online education offers the opportunity of introducing new technologies, which in general positively impact students’ perceptions and satisfaction, such as the perceived ease of use of e-learning platforms by students (e.g., “I find the e-learning system easy to use”) [24]. Artificial intelligence (AI), bots, big data, blockchain, robotics, barcode dispensing, management of patient health records, and computer-based support systems for decision making, among other innovations, are being introduced to pharmacy education [3,47,55,56]. Students are required to learn how to use these innovations during online education, which can also be applied to clinical settings. Overall, technological innovations are expected to revolutionize the future of pharmacy education, healthcare professions, and patient care [3].

#### 4.1.4. Online Classes/Training

E-learning can be used to provide online theoretical, practical, or other types of classes (e.g., storytelling classes), to record classes, to give access to documents and other materials, to publish grades, to provide exams, or to ensure communication between students, between students and professors, or between professors, among others. Thus, e-learning offers the opportunity of implementing more flexible programs [26,29,30,42,52,55]. Online classes and training sessions should be designed to ensure students’ knowledge acquisition and improve students’ skills and competencies, such as the use of technologies to help patients in managing their therapeutic regimen (safety and efficacy issues) [3].

During online training, pharmacy students have the opportunity to participate in virtual teleconsultations, real online practices, or consultations involving simulated patients, with the incorporation into patient care and the teaching–learning process [40,47]. Online pharmaceutical consultations may benefit from access to patients’ clinical data, which can raise confidentiality or data security issues [51,56]. For instance, virtual objective structured clinical examinations (OSCEs) promoted the acquisition of knowledge and competencies through real-life situations, with positive student opinions [45]. The positive aspects of OSCEs included schedule flexibility, decreased levels of anxiety, a higher sense of security, improved knowledge, satisfaction, communication, and better patient counseling (e.g., diabetes management, dispensation of over-the-counter medicines, warfarin initiation, or self-care aspects) [22,28,36,40,45].

Positively, online classes were classified by students as more flexible and versatile and less time-consuming since students could set their own pace (better time management) or spent less time traveling [10,52]. Many pharmacy students manifested a positive opinion about the diversity of online resources, such as forums, quizzes, videos, or games [32,33]. Students’ acquisition enforcement was also pointed out as an advantage since pharmacy students can choose when to see recorded classes: lectures or workshops [26,55]. Practice readiness was also classified as a positive issue, regarding the easy implementation of digitizing scoring systems or the facility of contacting teachers or colleagues or receiving scores and tasks through different applications [37,50,55].

#### 4.1.5. Faculty/School of Pharmacy and Other Variables

The availability of technical support by schools (e.g., hardware/software assistance); the possibility to receive interprofessional training, join in internships or postgraduation courses, and participate in continuing education during e-learning classes; and the chance to be involved in the development, evaluation, or validation of e-learning (e.g., team debates, focus groups) were among the variables related to pharmacy undergraduates’ positive perceptions [23,29,35,41,47,48,52,57].

### 4.2. Weaknesses

The weaknesses related to the internal environment were grouped into four groups: (1) students—human interactions and communication; (2) students—other; (3) online classes; and (4) technologies.

#### 4.2.1. Students—Human Interactions and Communication

In general, students reported dissatisfaction regarding the lack of communication, networking, and human interactions with their teachers, colleagues, patients, experts, and others [26,48,54,55]. Absent or decreased interactions contributed to students’ social isolation, missing colleagues, limited development of social skills, students’ negative emotional responses (e.g., less positive attitudes), and possible communication barriers during virtual learning (e.g., lower predisposition to clarify doubts or to ask/reply to questions) [26,44]. Thus, the acquisition of new communication skills (e.g., eye contact or talking slowly) seems to be essential to communicate online (e-communication) with peers or patients. Specific communication competencies are needed in the healthcare professions, such as promoting students’ consciousness, empathy, culture of care, or humanity, while respecting patients’ rights, for instance, during digital pharmaceutical consultations [47].

#### 4.2.2. Students—Other

Some pharmacy students classified e-learning as not effective or helpful [8,13,21,39,43,57], which confirms that online education can present some constraints/limitations. For instance, less knowledge acquisition was reported with e-learning in comparison to in-person sessions in some studies [11,12]. These findings confirm the need to regularly check the quality and effectiveness of online education in all schools of pharmacy.

The risk of cheating in online assessments was an underreported issue [23,55] which should be carefully evaluated in future studies. In particular, assessments based on closed-ended questions (i.e., when students can choose one (or more) correct options from a group of statements/predetermined answers) should be carried out in person to avoid the possibility of cheating. Additionally, students can be required to declare the non-use of artificial intelligence (AI) tools when elaborating monographs, theses, or other written works.

The worst e-learning experiences were reported by students with a lack of past experience in using online tools or limited digital literacy. The students’ difficulties in using digital technologies can be justified by poor digital literacy, a lack of active participation in the digital society, or living in a developing country (digital divide) [43,44,47,54]. Ideally, students’ digital skills should be evaluated before the provision of online education to ensure that digital tools are effectively used.

#### 4.2.3. Classes

There was an eventual degradation of the quality of education, with some students declaring being unsatisfied and less motivated. For instance, students’ acquisition of knowledge, skills, and competencies was negatively affected in some studies (e.g., practical and laboratorial classes) [39,53,55]. In general, the provision of lab classes was recognized as more difficult/complex online than in-person, although some strategies can be adopted, such as the use of recorded videos to demonstrate key practices, submission of videos, role-plays, or using the MyDispense software [23,27].

Pharmacy students have identified diverse issues related to e-learning methodologies, such as complex e-learning materials, the inexistence of instructions/norms, unfair or complex assessments, and impractical evaluation platforms, among others [48,55]. Thus, specific training to optimize the administration or reception of online courses/classes may be necessary [47]. Teachers should clearly explain, through oral and written instructions, the access to materials and how classes and assessments will be carried out online. Additionally, the usability and intelligibility of online platforms should be pretested by students and teachers (e.g., simulated students’ assessments). These procedures are expected to increase students’ comprehension of online assessment methodologies, regarding the quality of online assessments seems to have an impact on students’ satisfaction.

#### 4.2.4. Technologies

Students reported dissatisfaction with low speed and instability of the internet or other connection issues, besides the need for appropriate hardware, software, and internet, which implies monetary investments [27]. Thus, the provision of technical support by faculties is essential to ensure the appropriate implementation of e-learning sessions [57].

Additionally, some pharmacy undergraduates manifested e-learning disapproval if social networking tools were not used (e.g., “avoiding commonly used online tools such as YouTube and Facebook by instructors”), if the used e-learning tools were too complex, or if electronic devices such as smartphones or tablets were not used [48,58]. Students perceived the usefulness or awareness of online platforms as essential to their acceptance of e-learning [21]. The use of advanced and usable computer-based platforms, with the offer of an e-learning environment similar to face-to-face teaching, was preferred (e.g., ZOOM) [22,43,44,55]. Preferably, usability testing of online education systems should regularly be evaluated and monitored by students and teachers.

### 4.3. Opportunities

The opportunities related to the external environment were grouped into two groups: (1) students and (2) classes.

#### 4.3.1. Students

Students pointed out as advantages that they could access classes anywhere or living away from educational institutions, with saved time and reduced costs [48,55]. E-learning is more economical and sustainable than in-person classes (e.g., digitization of materials or an ecological footprint). In general, students felt improved family conviviality and comfort, with a likely better quality of life at home [33,44].

#### 4.3.2. Online Classes

E-learning seems to be a more immediate educational methodology. For instance, it ensures quicker contact with teachers or other academic staff through proper communication online channels (e.g., chats) or permanent access to online materials everywhere (24 h days) [33,55]. Additionally, online education offers the chance to receive external training from private or public health authorities, community or hospital pharmacies, regulators, the pharmaceutical industry, or other organizations, with a likely higher diversity of courses and content. These courses can cover wide audiences without space constraints, if necessary.

### 4.4. Threats

The threats related to the external environment were grouped into three groups: (1) students’ well-being, (2) classes, and (3) technologies.

#### 4.4.1. Students’ Well-Being

There is an increased risk of pharmacy students’ psychological problems (e.g., online exam phobia, frustration, anxiety, worries, or stress) or physical disorders/well-being compromise (e.g., posture and vision issues due to long-time screen use) [21,31,34,49,55]. Stress can be justified by students’ psychological distress, inexperience, and unpreparedness, regarding online education [21]. Academic uncertainty, job insecurity, and dissatisfaction with e-learning activities can negatively affect students’ mental health. Fewer social interactions with colleagues outside of classes may also justify students’ dissatisfaction, social isolation, and frustration [31].

Students are more likely to be distracted at home, which may be due to the presence of family members (or other persons) or a higher opportunity for leisure activities [8,13]. There is low resistance to tobacco or unhealthy foods because students can become more anxious at home [31]. The obligatory acquisition of a laptop/tablet or the internet bill increases costs, which may explain pharmacy students’ dissatisfaction in some cases [22].

#### 4.4.2. Classes

The presence of other family members at home may raise confidentiality issues (e.g., pharmaceutical teleconsultations) and/or interfere with students’ concentration and activities during classes. Internet connectivity issues may lead to the interruption of classes (e.g., disconnection, poor audio and/or video quality, or power cuts) [21,44], which reinforces the importance of technical assistance by schools/faculties.

#### 4.4.3. Technologies

Barriers to access to online education, especially for economically and socially vulnerable students or students with low digital literacy, were identified. For instance, restrictions in access to electricity, the internet, or digital technologies are more prevalent in developing countries (digital divide) [27,47,54]. One risk/threat of using digital technologies is cybercrime or cybersecurity issues. Thus, students should install proper antivirus software, which also increase costs.

### 4.5. Takeaways for Educators

Strengths and weaknesses should be carefully evaluated and optimized since they are directly (or indirectly) related to pharmacy students’ satisfaction, self-confidence, engagement, and/or motivation. Both strengths and weaknesses can be integrated into the strategic planning of faculties. For instance, more online classes may be created, with the use of visual aids, interactive materials, and/or new disruptive technologies (e.g., use of AI during pharmaceutical consultations or the access to online patient data/databases), regarding strengths [24,40,44,46,47,56]. Additionally, potential weaknesses should be carefully addressed. For instance, interactive tasks and group activities can be introduced during online classes to bridge the lack of human interactions and/or potential social isolation, the quality of e-communication and students’ knowledge acquisition or culture of care should be supervised since virtual interactions can be more problematic/limited than in-person interactions [11,12,47], technological issues (e.g., internet speed) should be solved, students with limited digital literacy should be identified and supported [27,43,44,47,54,57], and the needs of unsatisfied pharmacy students’ with online education should be carefully identified and addressed, for instance, through using regular quality queries.

Opportunities and threats should be individually analyzed by each faculty/school of pharmacy. If necessary, opportunities as well as the solutions for threats should be integrated into the strategic planning of faculties, since opportunities need to be strengthened (e.g., tailoring the number and type of online classes according to the needs of students) and threats need to be avoided (e.g., ensuring regular supervision of students’ well-being or the availability of adequate technological means for the less favored students).

### 4.6. Reply to Research Question and Practical Implications

Some students declared lower levels of knowledge acquisition, satisfaction, and psychological well-being with online education when compared to face-to-face teaching [8,13,21,26,54], which seems to support/indicate the need for optimizing e-learning methodologies. For instance, older adult learners may have more difficulties in the adoption of e-learning than younger students because the first may be less familiar with digital technologies [59].

The present narrative review exhaustively systematizes groups of strengths, weaknesses, opportunities, and threats regarding the perspective of pharmacy students on e-learning, which can be used to develop new guidelines or to evaluate e-learning practices in pharmacy schools. It is important to notice that previous research on the present topic is limited. As in the case of face-to-face teaching, online education must be exhaustively evaluated (e.g., the maximum number of variables with possible impact on pharmacy students’ perception about e-learning can be analyzed).

The development of national and international guidelines about online teaching/e-learning of health sciences, such as pharmacy is recommended. For instance, to achieve or maintain: a proper format and quality of materials (e.g., interactive materials), a correct e-communication, a suitable teacher performance, functional students’ assessments, adequate students’ psychological well-being, a reasonable acquisition of knowledge or skills, acceptable practical classes (e.g., pharmacy practice) or laboratorial sessions, satisfactory teleconsultations, or an appropriate acquisition of laboratory skills/competences [13,47,60,61]. Future guidelines about e-learning should discuss how to overcome weaknesses and threats as well as how to ameliorate/reinforce strengths and opportunities, respectively [62]. Additionally, strengths and opportunities can be used to overcome weaknesses and/or threats. For instance, podcasts about how to use e-learning platform can be used to teach/motivate new students or pharmacy students with limited digital literacy and technology experience.

SWOT analysis can be helpful to design, to manage, or to monitor/supervise the quality of online education. Online questionnaires or face-to-face interviews can be used in the regular monitoring of the quality of online education (e.g., groups of students randomly or conveniently selected). Student’s opinions and feelings are more likely to be collected in detail in interviews or focus groups, with open rather than closed-ended questions [13]. The development of human competencies and skills to operate technologies (e.g., patient-centered e-communication in classes or teleconsultations) should be integrated in the strategic plans of faculties [47,61], which can be less focused on human factors.

Besides the creation of new guidelines on e-learning for pharmacy curricula, SWOT analysis can contribute to strategically ensure the quality of online education or to develop a paradigmatic model of online education in health sciences, such as pharmaceutical sciences. SWOT findings should be regularly collected and integrated in faculty’s vision, mission, and strategic planning.

In general, the identified studies were much more focused on strengths and weaknesses than in opportunities and threats, which seems to demonstrate the need of developing additional research on these areas. Thus, eventual research gaps on the present topic need to be addressed in future research. Examples include pharmacy students’ perception of the provision of e-learning by external experts and acquisition of skills, namely in practical classes or knowledge assessments due to the possibility of cheating.

### 4.7. Limitations and Strengths

Considering that the present narrative review was carried out by just one author, data collection, summary, analysis, discussion, or quality assessment were not examined by peers. Additionally, the present SWOT analysis could have been influenced by the fact that almost all the selected studies were carried out during the COVID-19 pandemic. For instance, students’ mental state could be negatively influenced by the pandemic. Thus, future confirmatory research is recommended. However, at least some similar/common findings have been found in previous research about online e-learning for undergraduates in health professions (e.g., dentistry, nursing, physical therapy, and pharmacy students), such as higher knowledge or skill gains with e-learning when compared to in-person classes [63,64].

The individual quality of the selected papers was not specifically evaluated through the application of quality tools [65]. The only quality indicator was based on the peer review of the selected papers. Despite the use of a saturation methodology to conclude/close the analysis of the selected papers/works, additional databases, and keywords (e.g., virtual) are recommended in future research. The development of a systematic review and meta-analysis is recommended for future research because more narrow methodological elaboration criteria are followed.

The limited number of published SWOT analyses on the present topic and the exponential increase in published studies about the perceptions/opinions of pharmacy students on e-learning during the COVID-19 pandemic are among the study strengths. The present narrative review broadens the state of the art on the present topic and, necessarily, includes more details about pharmacy undergraduates’ opinions/perceptions on e-learning.

## 5. Conclusions

A SWOT analysis of pharmacy students’ perspectives on e-learning was developed, which can be used in the optimization of online classes or in the strategic planning of digital education in pharmacy schools, as well as, in other higher education institutions. Overall, e-learning methodologies seem to be suitable for pharmacy students, with an expressive number of identified strengths (e.g., students’ engagement, acceptance, motivation, satisfaction, or other positive perception) and opportunities (e.g., location flexibility). However, some ameliorations are required to resolve the identified weakness (e.g., less in-person patient care experience or limited conditions for providing laboratory classes) and threats (e.g., higher risk of psychological problems, anxiety, stress, depression, low resilience, loneliness, social isolation, and frustration).

## Data Availability

No new data were created.
